# Manual of Practical Midwifery; Containing a Description of Natural and Difficult Labours, with Their Management. Intended Chiefly as a Book of Reference for Students and Junior Practitioners

**Published:** 1836-04

**Authors:** 


Art. VII.—*Manual of Practical Midwifery; containing a Description of Natural and Difficult Labours, with their Management. Intended chiefly as a book of reference for Students and Junior Practitioners. Illustrated by fifteen engravings*. By James Reid, m.d. &c. London. 1836. 12mo., pp. 246.

Dr. Reid tells us in his preface that he has frequently heard students, and those just entering on the arduous practical duties of the accoucheur, complain of the want of a work, which, while it should include all the information that might be necessary in the moment of doubt and difficulty, could still, from its size, be portable, and easily referred to at the precise time when assistance is so anxiously required, and delay might be so perilous. To supply this assumed deficiency in our medical catalogue, the little volume before us is offered to students and junior practitioners. This announcement of the object the author had in view led us to expect he would pursue a totally different plan from that which he has adopted. We expected that the book would contain a series of aphorisms (like Denman's on the Forceps,) describing the nature of the difficulties that are most likely to perplex a practitioner of midwifery, who is to carry his 12mo. guide in his pocket, with general rules as to the treatment required in such cases. Dr. Reid runs slightly and superficially through the anatomy of the pelvis; precursory symptoms of labour; duties of the accoucheur, and most of the subjects connected with parturition which are to be found in elementary treatises on midwifery; but we look in vain, and so will the practitioner, "at the precise time when assistance is so anxiously required, and delay might be so perilous," for brief and clear directions as to the management of doubtful and dangerous cases. The student who wishes, before he commences his obstetrical studies, to run over the enumeration of the leading facts connected with them, may, without much loss of time, look over this little volume. With every disposition to be indulgent, we cannot conscientiously say more in its praise, or recommend it to any other class of readers. We must suggest to Dr. Reid the general propriety of referring to English works when they are quoted by foreign writers. If he had consulted Dr. Merriman, he would have found that 48 cases of convulsions are *not* stated as having occurred in 2000 labours. In 2947 cases attended by Dr. Merriman, only five were complicated with convulsions. The remaining 43 were *consultation* cases; and, consequently, no average of the general frequency of the disease could be drawn from them. In order to correct M. Velpeau's mistake, of which, indeed, Dr. Reid hints his suspicion, Dr. Merriman published a letter in the Edinb. Med. and Surg. Journal for January, 1835.

We admit that elementary works, when well compiled, are in general useful. They prepare a student for his studies; and serve as references or synopses for him to look over, for the purpose of recalling and impressing upon his memory the more elaborate statements of his lecturer; but this we regard as the extent of their utility; and we altogether disapprove of them as pocket guides for ignorant students or inefficient practitioners in the moment of peril: from such assistance an ignorant man may, indeed, be made bolder, but therefore more dangerous; for he may be tempted to rely upon himself and his horn-book, when without this imaginary help, a consciousness of his own deficiency would prompt him to seek counsel and aid from another and more trust-worthy practitioner.

